# Small-scale field assessment against the dengue vector *Aedes aegypti* using the auto-dissemination approach in an urban area of Vientiane, Lao PDR

**DOI:** 10.1371/journal.pone.0270987

**Published:** 2022-07-01

**Authors:** Phoutmany Thammavong, Sebastien Boyer, Phonesavanh Luangamath, Nothasine Phommavanh, Vaekey Vungkyly, Somphat Nilaxay, Khaithong Lakeomany, Paul Brey, Marc Grandadam, Sebastien Marcombe

**Affiliations:** 1 Medical Entomology and Biology of Disease Vectors, Institut Pasteur du Lao PDR, Ministry of Health, Vientiane, Lao PDR; 2 Medical and Veterinary Entomology Unit, Institut Pasteur du Cambodge, Phnom Penh, Cambodia; Fundacao Oswaldo Cruz Instituto Rene Rachou, BRAZIL

## Abstract

**Background:**

In Lao PDR, dengue fever is the most important vector borne disease and vector control remains the principal method to fight against *Aedes aegypti* the primary transmitter mosquito species. Vector control management programs need new strategies in addition to conventional larviciding and adulticiding interventions in the country. In this study, we examined the In2Care® Mosquito Trap’s efficacy using insecticide auto-dissemination strategy. The insecticide pyriproxyfen, present in powder form inside the trap station, contaminates the body of gravid female mosquitoes visiting the traps and is later on disseminated via the mosquitoes in breeding sites surrounding the traps. We tested the attractiveness of the Traps, their efficacy to reduce the larval and adult abundance, and the impact on emergence rates. Specifically, we tested if the servicing interval of the In2Care® Mosquito Trap could be extended to 12 weeks.

**Methods:**

Two black plastic ovitrap buckets and two BG® sentinel traps were placed in the premises of the Science campus of Vientiane Capital located in an urban area to measure weekly the larval and adult relative abundance of *Aedes* mosquitoes from 2017 to 2019. Twenty-five In2Care® Mosquito Traps were evenly distributed in this area and two studies of 12 weeks were implemented during January and April 2018 and, July to October 2018 (dry and rainy season, respectively). Every 2 weeks, water samples from 5 In2Care® Traps were randomly selected and tested at the laboratory with *Ae*. *aegypti* larvae to measure the larval and pupal mortality. The relative abundance of *Aedes* mosquitoes in the BG traps® with the presence of In2Care® Traps in 2018, was compared with the surveillance results obtained in 2017 and 2019 without In2Care® Traps. Every week, water samples from the ovitrap buckets were tested for Emergence Inhibition (EI).

**Results:**

The In2Care® Traps were very attractive to gravid *Ae*. *aegypti* mosquitoes specifically during the rainy seasons with 96% of the traps colonized with larvae/pupae within four weeks. The bioassays showed 100% mortality in the water samples from the traps during the twelve weeks studies showing the good efficacy over time of the pyriproxyfen without additional servicing in the 12 week period. In addition, the larvicide was successfully disseminated into the ovitrap buckets placed in the treated area where 100% of EI during all weeks of intervention was measured. There was no significant effect of the treatment on adult abundance reduction in the treated area, probably due to recolonization of adult mosquitoes surrounding the field experiment.

**Conclusions:**

The observed potential of the In2Care® Mosquito Trap using the auto-dissemination strategy could lead to the use of this new tool in combination with conventional control methods against Dengue vectors in urban tropical areas. Large scale field trials should be implemented in Lao PDR to prove its efficacy for Public Health programs.

## Introduction

In Lao PDR, since the 1980s, dengue fever re-emerged annually in a way that brought the disease as a major public health concern, mainly in urban areas [1–3]. Between 2013 and 2019 the estimated annual cases varied between 2,000 and 44,000 [4]. The country also faced two major outbreaks in 2013 and in 2019 with respectively 44,000 and 38,000 suspected cases recorded [4]. The disease is transmitted to humans mainly by the mosquito *Aedes aegypti* and to at a lower extent by *Ae*. *albopictus* [5]. The disease control is mostly focused on targeting the vectors as no enough effective vaccine or specific treatments are available. During dengue epidemics, control programs rely on the use of pyrethroid insecticides (i.e. permethrin and deltamethrin) to reduce adult mosquito populations [2]. However, this method is threatened by the development of insecticide resistance in *Aedes* mosquitoes in most parts of Lao PDR [2]. To reduce *Ae*. *aegypti* or *Ae*. *albopictus* larvae populations, Lao PDR has relied on the use of the insecticide temephos (Abate® formulation, organophosphate family). Recently, the use of temephos was replaced by the bio-larvicide *Bacillus thuringiensis israelensis* (*Bti*). Both insecticides are used to treat large and known water containers and distributed throughout the country in places where dengue cases are reported [2]. However, *Ae*. *aegypti* and *Ae*. *albopictus* species also breed in cryptic habitats which are difficult to reach and inaccessible to direct treatments [6–9]. In order to target these hidden specific breeding sites, the auto-dissemination approach may be a mean to be used by exploiting adult mosquitoes as vehicles of insecticide transfer to disseminate in oviposition sites [10]. Several studies showed that auto-dissemination is a promising method [10–13] and can effectively reduce *Aedes aegypti* and *Ae*. *albopictus* mosquitoes number in laboratory and field settings [8, 10, 11, 14–18]. The insecticide pyriproxyfen (PPF) is a bio-pesticide (insect growth regulator, IGR) which does not cause direct adult mosquito mortality [19], but aims immature stages, specifically at the pupal stage. In2Care® Mosquito Traps using this auto-dissemination strategy were developed to target dengue vectors [20–22]. It acts at very low concentrations compared to conventional larvicides on both *Ae*. *aegypti* and *Ae*. *albopictus* [1, 8, 23–25]. Itoh *et al*. [7] showed that female mosquitoes can acquire crystals of PPF by landing on a treated surface and later lay the insecticide in surrounding breeding sites that they later visit. The In2Care® Trap lures and contaminates *Aedes* mosquitoes with PPF and a fungal adulticide, *Beauveria bassiana* that slowly kills contaminated adult mosquitoes after a few days [20, 21].

Spinosad is a biopesticide composed of a mixture of two metabolites (spinosyn A and D) produced by the soil bacterium *Saccharopolyspora spinosa* (Actinomycetes), specifically active against larvae mosquitoes and has been found to have low toxicity for humans and other non-target fauna [26] and has potential to be used against mosquitoes as it does not show cross-resistance with conventional insecticides [27]. Research into new strategies aimed at limiting the development of insecticide resistance in mosquitoes has been orientated towards the use of mixtures of two insecticides, where each one possesses a different mode of action. In the laboratory, the association of pyriproxyfen and spinosad has been found to be synergistic against *Ae*. *aegypti* larvae and in reducing the number of emerging adults [28]. In our studies we tested the residual efficacy of the mixture of spinosad and pyriproxyfen in five In2Care® traps.

The objectives of the present study were to assess the In2Care® Mosquito Trap’s attraction and its larvicidal and auto-dissemination impacts over twelve weeks against *Ae*. *aegypti* mosquitoes in a small area of Vientiane capital, using bioassays and relative larval and adult abundance surveillance from 2017 until 2019. Also, this experiment was part of a pre-evaluation study to be executed in Lao PDR before the onset of the ECOMORE2 project large-scale field trial (600 traps in two urban areas of Vientiane during a year; https://ecomore.org/).

Indeed, the secondary objective of the study was to test the various technical aspects of this strategy with the help of this specific trap (e.g. installation, servicing, staff training etc.) under Laotian conditions (high temperatures and humidity).

## Material and methods

### In2Care® Mosquito Traps

The In2Care® Mosquito Traps and In2Mix® sachets (In2care, Wageningen, The Netherlands) containing 74.03% PPF and 10% *B*. *bassiana* strain GHA, as active ingredients, were used for the experiments [23, 24]. The trap is a black polyethylene pot of 27 cm wide and 18 cm deep and holds a maximum of 5 liters of water (https://www.in2care.org/mosquito-trap/). A 5-cm-wide piece of static netting treated with In2Mix® (consisting of PPF particles and *B*. *bassiana* spores) is wrapped around a floater which is placed on the water surface. The water is then treated with the left over In2Mix® from the sachet. For optimal efficacy, the manufacturer recommends that the traps are serviced every 4–6 weeks, replacing the powder-treated static netting with a new one and adding water. In this study, we looked at the possibility of extending the servicing time to 12 weeks, by not replacing the treated netting within 12 weeks.

Spinosad tablets containing 7.48% of spinosyn A and D mixture (Natular® DT, Clarke Mosquito Control Product, Inc. Roselle, IL, USA) were used to treat the five randomly selected In2Care® Mosquito Traps

### Study site/Experimental set up

**Fig 1:**
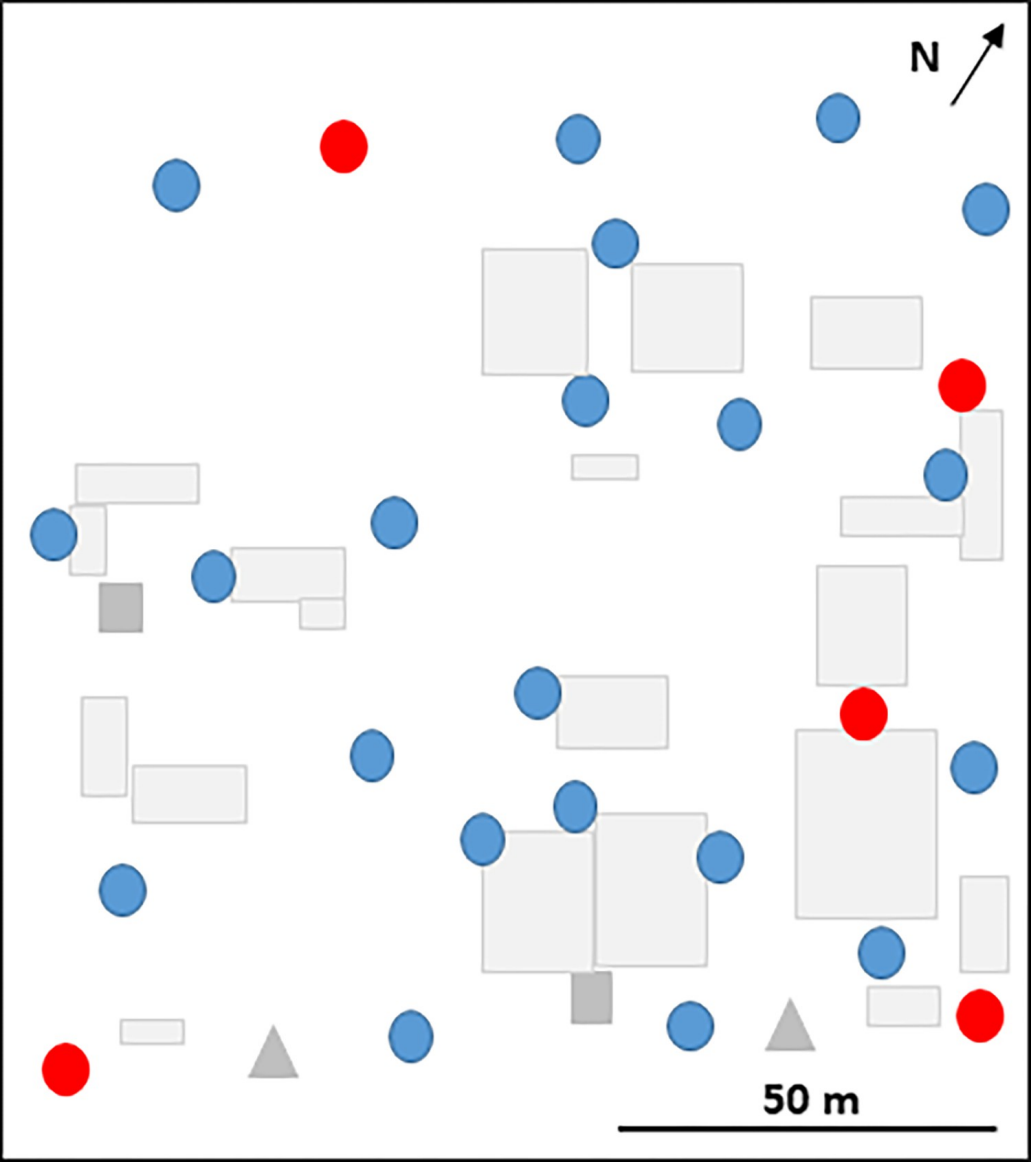
Locations of the 25 In2Care® Mosquito Traps in the science campus, Vientiane Capital, Lao PDR, 2018. Traps with In2Mix® (blue dots; n = 20), traps with In2Mix® and spinosad (red dots; n = 5), BG sentinel traps (grey square; n = 2*)*, and bucket ovitraps (grey triangle; n = 2).

Our study was conducted in the premises of the scientific campus of Vientiane capital, (17.962684°N, 102.615035°E) composed of the Institut Pasteur du Laos (IPL), Centre of Infectiology Lao Christophe Mérieux (CILM), University of Health Science and the Food Safety Laboratory of Lao PDR (Fig 1). This area is an urban zone, including trees and bushes, and the campus is surrounded by a bus station, a fresh market, shopping malls and, government offices. Twenty-five In2Care® Mosquito Traps were installed in dry and vegetated and/or shaded locations in this 1.6 ha area, distributed, every twenty meters representing 1 trap per 400m^2^ as recommended by the manufacturer (Fig 1). Among the twenty-five traps, five of them were chosen after a draw and treated with spinosad (mixture of spinosyn A and D, Natular® DT Larvicide Tablets [7.48%], Clarke, St. Charles, IL, USA) at the beginning of the study (Fig 1). Two studies were carried out, during the dry season from January to April and in the rainy season from July to October, in the year 2018. The In2Care® Mosquito Traps were placed at the same locations both seasons and the spinosad treated traps as well.

### Mosquito surveillance and emergence rates in ovitrap buckets

Relative adult and larval abundance of *Aedes* mosquitoes were surveyed weekly between 2017 and 2019 in the experiment area. For this, two BG sentinel® traps and two black plastic ovitrap buckets (5 litres of water without attractant) were used. The water levels in the buckets was topped up when needed. Emergence inhibition was also measured in 250 mL cups containing the water samples and the larvae/pupae from the buckets. The emergence rate was compared to control cups filled with untreated water and 25 larvae (L3-L4 stage) of the IPL *Ae*. *aegypti* strain described in Marcombe *et al*. [1]. The adult mosquitoes that emerged from the bucket water samples and the adults from the BG traps were identified in the laboratory to measure the proportion of *Ae*. *aegypti* and *Ae*. *albopictus*.

### In2Care® Mosquito Trap insecticide residual efficacy and water level

The traps were placed at the exact same selected locations for the two studies. After 4, 6, 8, 10 and, 12 weeks post-deployment, we measured the water levels and visually, the water quality (presence of leaves, other animals, turbidity, and smell) in the traps (S1 Table). The water level was measured by the height of the water in the trap. For the first study only (dry season), at week 6, the water was topped up to five liters in all traps. We also recorded the number of *Aedes* eggs, and alive and dead larvae/pupae (S1 Table). At every monitoring time-point, five traps (4 In2Mix®-treated traps and 1 In2Mix® and spinosad-treated trap) were randomly selected and removed to test the larvicidal efficacy of the treated-water.

At every monitoring time-point, the residual efficacy of In2Mix® and In2Mix® with spinosad was tested in the laboratory. If the water samples (250 mL) from the traps did not contained *Aedes* larvae, twenty-five larvae from the *Ae*. *aegypti* IPL strain were added to measure the emergence rate and larval/pupae mortality. Otherwise we measured the mortality of the larvae/pupae already present in the traps. The results were compared to control cups filled with untreated water with 25 IPL strain larvae (L3 to L4 stages).

### Statistical analysis

The mosquito surveillance data were collected with two ovitrap buckets and two BG traps from January 2017 until December 2019. The data of both trial periods collected in 2018 were compared with the data in the same months in 2017 and 2019 analyzed with an ANOVA with Tukey post-hoc analysis in R (R Development Core Team, 2020) using the “car” package. For all statistical analyses, the significant *P* value was set at 0.05 or less.

## Results

### Trap attractiveness and water level

**Fig 2:**
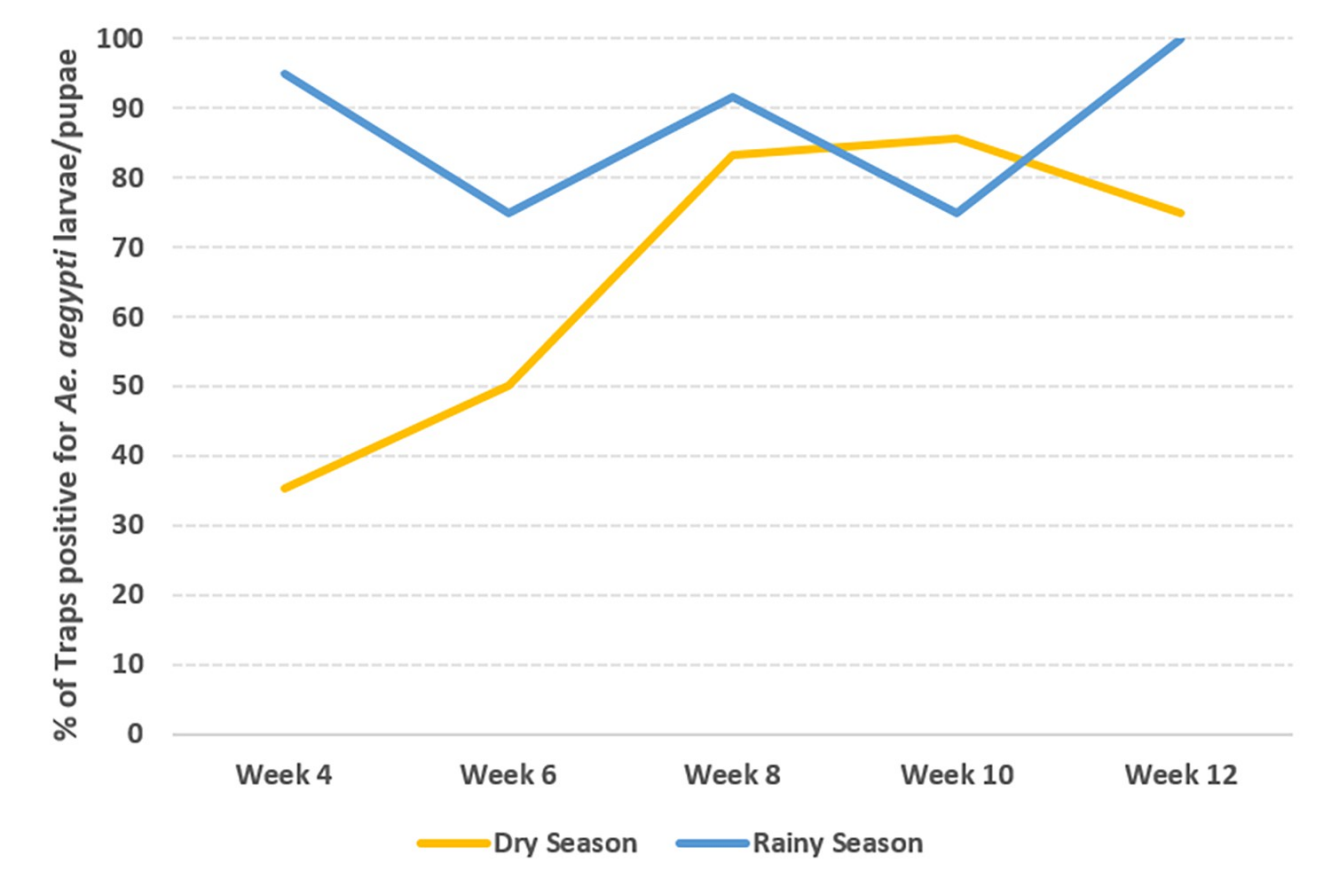
Percentage of In2Care® Mosquito Traps with In2Mix® positive with *Aedes* larvae during the dry and rainy seasons (Yellow and blue lines, respectively). The traps treated with spinosad were not included in colonisation calculation as this insecticide kills the first stage larvae.

During the rainy season, the colonization by *Aedes* mosquito was in 95% of the traps within 4 weeks and at every time point more than 75% of the traps were positive with *Aedes* larvae during the 12 weeks study. We observed a slower colonization during the dry season with only 30% positive traps within 4 weeks and within 8 weeks 83% of the traps were positive (Fig 2).

**Fig 3:**
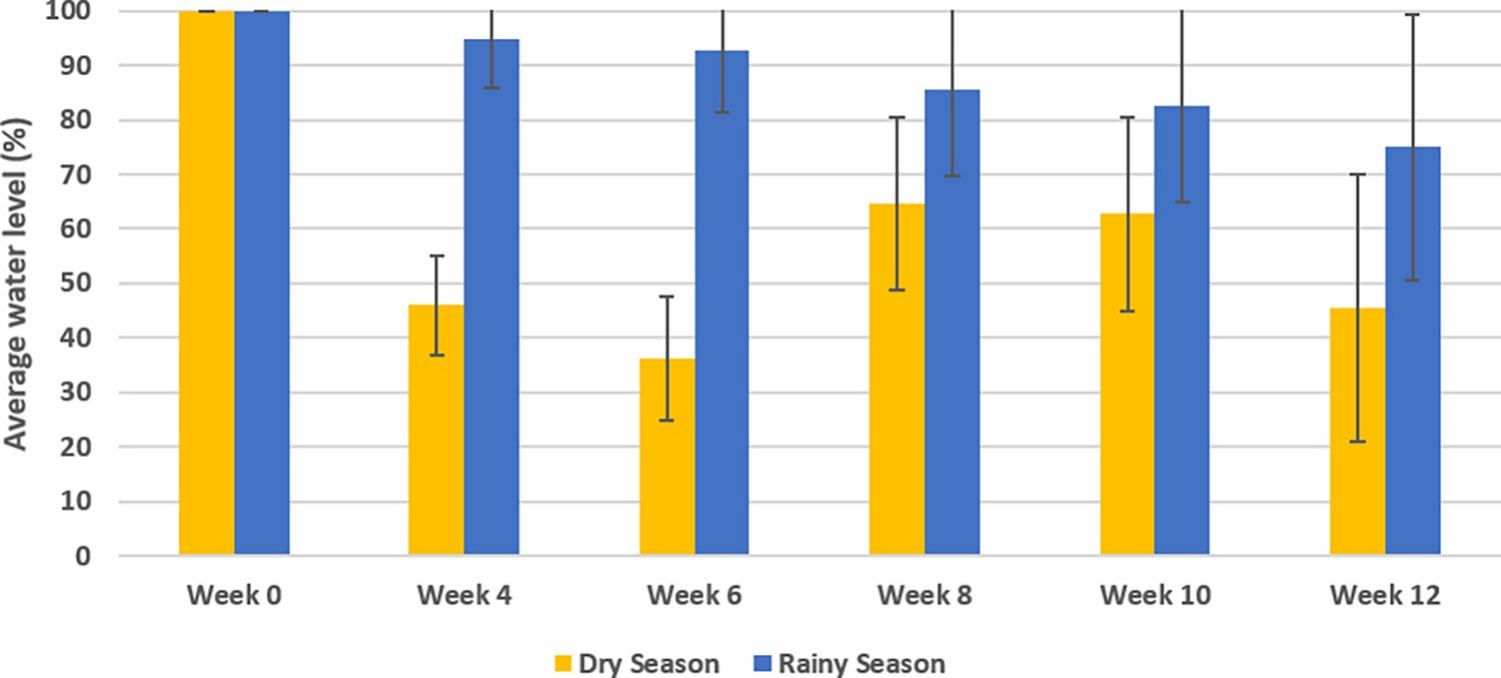
Average water level (%) in In2Care® Mosquito Traps with In2Mix® during the dry (yellow bars) and rainy (blue bars) seasons. Confidence intervals are one standard deviation of the mean.

Results showed a faster decline in water content during the dry season compared to the rainy season (Fig 3). During the dry season, water in three traps completely evaporated within 4 weeks and another 2 traps were dry at 6 weeks. The dry traps were refilled when checked. All traps were refilled up to 5 L (= 14cm) water at 6 weeks in dry season. During the first trial in the dry season, the water levels in the traps decreased from 14 cm (5L) to 6.2 cm (<2.2L) on average (±SD 3.8cm) with several almost empty traps (<4 cm) after 6 weeks (Fig 3). After the water top up at week 6, the average water levels varied from 9.9 cm (±SD 1.4 cm) to 6.9 cm (±SD 1.2cm) between week 8 and week 12, respectively. During the rainy season water was not topped-up. The average water level in the traps decreased but was still higher than 10 cm after week 6 (±SD 1.9cm) and 12 (±SD 2.9cm) (Fig 3).

### Residual efficacy of pyriproxyfen and spinosad in the In2Care® Traps

During the 12 weeks study, for both trials, there was no emergence of *Ae*. *aegypti* from the water samples from the In2care® Mosquito Traps treated with PPF only and PPF with spinosad while emergence in controls varied from 80 to 100% (Table 1). The larval mortality during the dry season in water samples from traps treated with PPF varied from 10 to 52% (average of 31%±16.2) and the pupal mortality was between 48 and 90% (average of 67.2%±17.6) showing the better killing activity of PPF against pupae. In the traps treated with spinosad the larvae never developed into pupae with a larval mortality of 100%. During the rainy season, the PPF insecticidal efficacy was higher against larvae (19 to 87% mortality; average 58.9%±23.7) compared to pupae (13 to 81%; average 41.1%±23.7). Even higher mortality (84 to 100%) was observed at the larval stage (similarly to the dry season) with traps treated with PPF and spinosad.

**Table 1:** Percentage of larval and pupal mortality and adult emergence (average) in water samples collected in In2Care® Mosquito Traps every 2 weeks. (In2Mix®: pyriproxyfen [PPF] and *Beauveria bassiana*; SPD: spinosad).

Trial	Week	Insecticide	% Larval mortality	% Pupae mortality	% emergence
**1**	Week 4	PPF	37.62	62.38	0
**Dry season**		PPF + SPD	100	no pupae	0
		Control	0	0	100
	Week 6	PPF	52.44	47.56	0
		PPF + SPD	100	no pupae	0
		Control	0	0	100
	Week 8	PPF	9.57	90.43	0
		PPF + SPD	100	no pupae	0
		Control	0	0	100
	Week 10	PPF	40.59	50.41	100
		PPF + SPD	100	no pupae	0
		Control	20	0	80
	Week 12	PPF	15	85	0
		PPF + SPD	100	no pupae	0
		Control	12	0	88
**2**	Week 4	PPF	54.12	45.88	0
**Rainy season**		PPF + SPD	100	no pupae	0
		Control	8	0	92
	Week 6	PPF	86.75	13.25	0
		PPF + SPD	100	no pupae	0
		Control	0	0	100
	Week 8	PPF	54.55	45.45	0
		PPF + SPD	84	16	0
		Control	4	0	96
	Week 10	PPF	79.59	20.41	0
		PPF + SPD	88	12	0
		Control	20	0	80
	Week 12	PPF	19.32	80.68	0
		PPF + SPD	100	no pupae	0
		Control	12	0	88

### Mosquito surveillance and emergence rate

The identification of the *Aedes* mosquitoes after emergence from the buckets showed that more than 98% of the mosquitoes were *Ae*. *aegypti* and the other two percent were *Ae*. *albopictus*.

**Fig 4:**
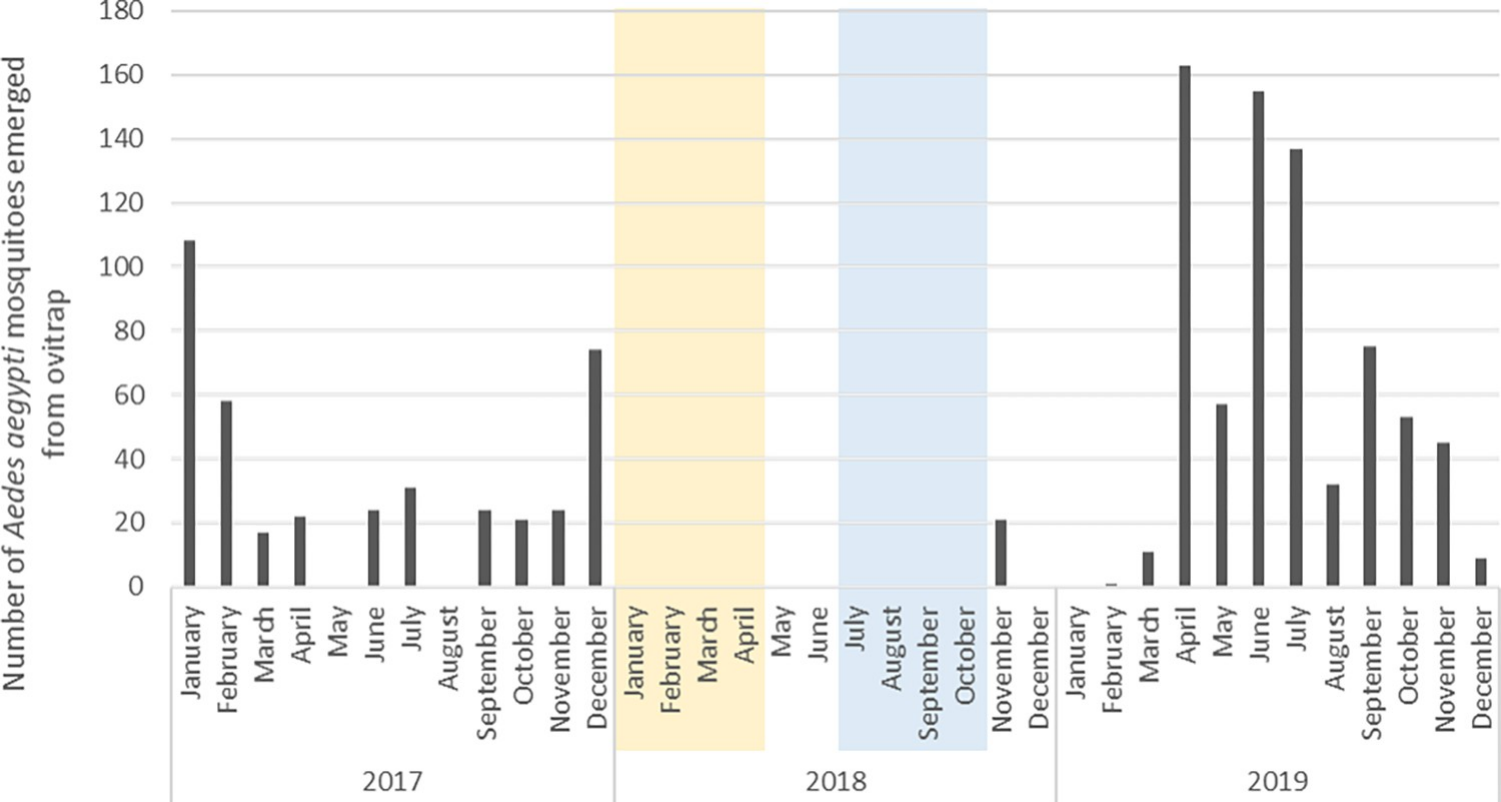
Number of *Aedes aegypti* mosquitoes emerged from the buckets from January 2017 until December 2019. Timelines of trial in dry season (yellow) and rainy season (blue) are indicated in the Fig.

**Fig 5:**
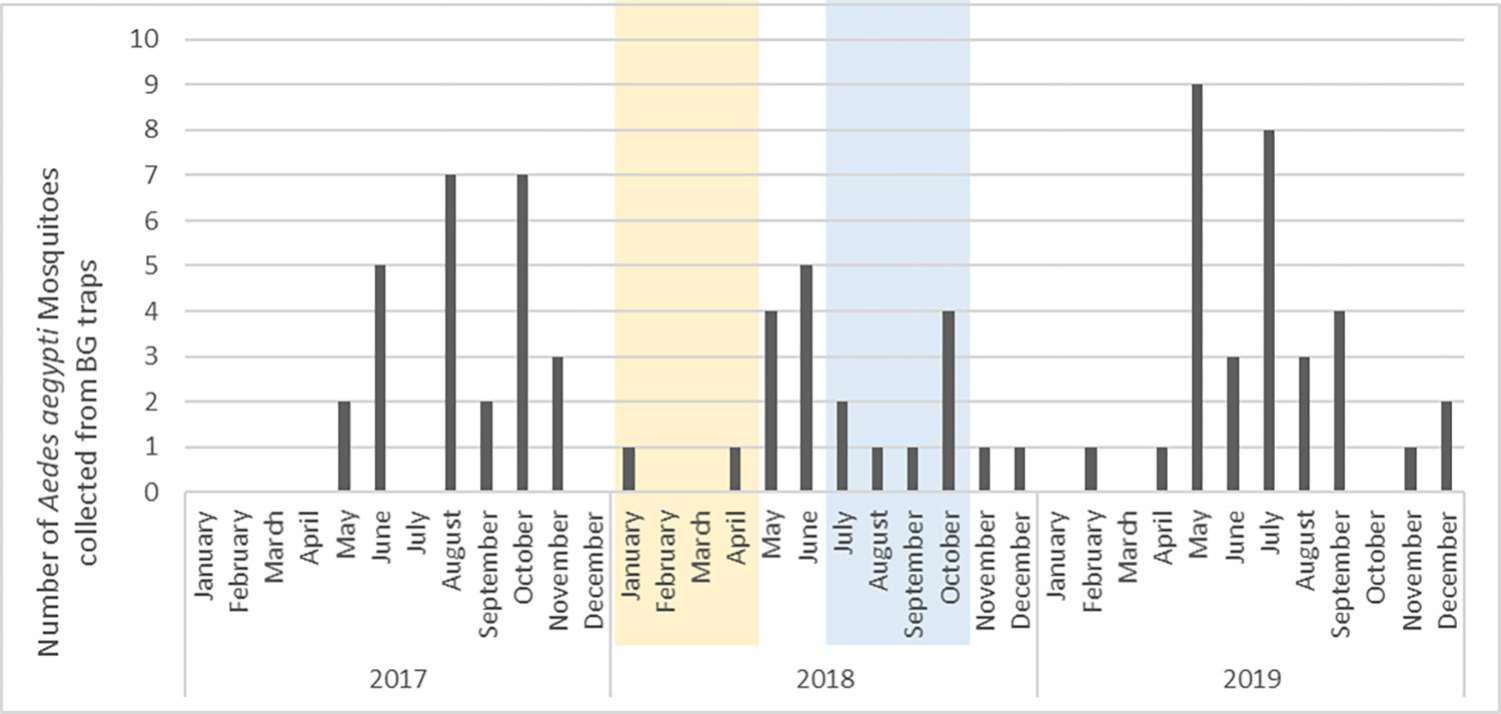
Number of *Aedes aegypti* adult mosquitoes caught in the BG traps from January 2017 until December 2019. Timelines of trial in dry season (yellow) and rainy season (blue) are indicated in the Fig.

**Fig 6:**
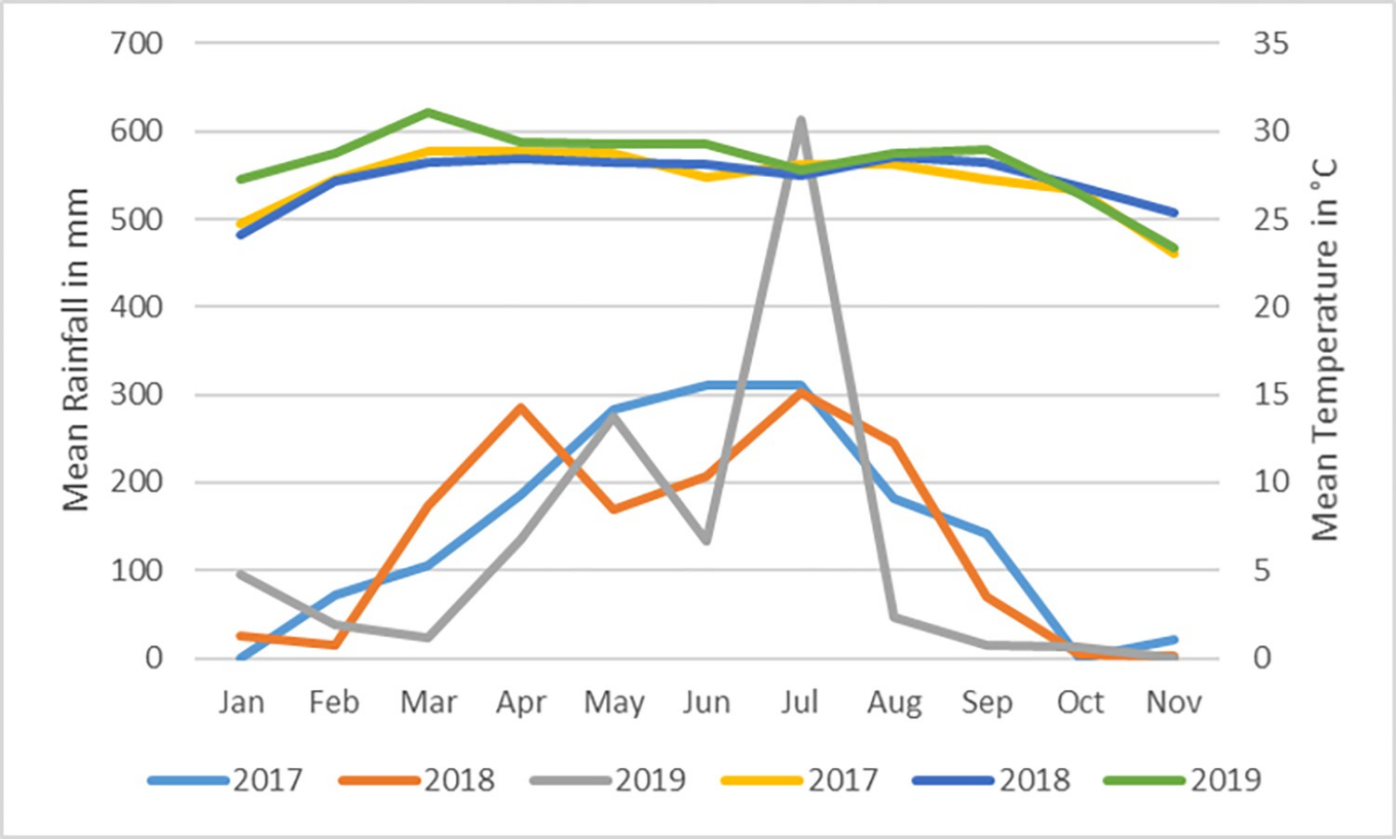
Mean precipitations (mm) and temperatures °C recorded in Vientiane capital between 2017 and 2019.

No mosquito emerged from the larvae/pupae caught in the buckets from January to October 2018, the period of the two trials (Table 2 and Fig 4). There was 100% emergence inhibition meaning that there was auto-dissemination of the PPF and that 100% of the caught *Ae*. *aegypti* larvae in the surrounding buckets were killed during the two trials. There was a significant difference found between emerged mosquitoes from the buckets in dry season of 2017 and of the intervention year 2018 (Table 2). In the rainy season there was a significant difference between the intervention year 2018 (less emergence), and the control years 2017 and 2019 (P < 0.05, Table 2). There was no significant difference between the mosquito catches with the BG-traps, in the periods before, during and after the trials compared to the same months of both the dry season trial (P = 0.585), and rainy season trial (P = 0.211, Table 3). However, on average there were less mosquitoes caught during the rainy season trial period in 2018 compared to the same period in 2017 and 2019 (not significant). In Fig 5, the number of mosquitoes caught in the BG-traps from January 2017 to December 2019 is shown. During the 3 years surveillance in the study sites, adult *Ae*. *aegypti* mosquitoes were caught almost every month and followed a typical seasonality abundance pattern with more mosquitoes caught during the rainy season. The Fig 6 shows higher rainfalls and lower temperatures in the rainy seasons compared to the dry seasons during the three years study.

**Table 2:** Average number (± Standard deviation) of *Aedes aegypti* mosquitoes emerged from buckets in 2017 and 2019, compared to the same period as the two trials in dry and wet season during 2018. P-values of ANOVA tests indicate that there is a significant difference between years (p < 0.05). Letters indicate significant difference.

	2017 (pre-intervention)	2018 (intervention)	2019 (post-intervention)	P -value
**Dry Season (January—April)**	34.2±20.3 a	0.0±0.0 b	21.9±31.5 ab	0.0467
**Rainy Season (July–October)**	9.5±10.5 a	0.0±0.0 b	33.0±35.8 a	0.0469

**Table 3:** Average number (± Standard deviation) of *Aedes aegypti* adult mosquitoes caught with BG Sentinel in 2017, 2018 and 2019 in same period as the 2 trials in dry and rainy season. P-value of ANOVA test indicate that the mosquitoes caught per year in the given periods are not significantly different (p >0.05).

	2017 (pre-intervention)	2018 (intervention)	2019 (post-intervention)	P -value
**Dry Season (January—April)**	0.0±0.0	0.3±0.5	0.3±0.5	0.585
**Rainy Season (July–October)**	2.4±2.2	0.9±0.8	1.7±1.8	0.211

## Discussion

The success of auto-dissemination strategy to reduce mosquito population abundance depends on four criteria; (i) attraction of mosquitoes to the stations (ii) kill the larvae and pupae in the traps (iii) transfer of chemicals to the mosquitoes and (iv) sufficient dissemination of chemicals to target surrounding breeding habitats. In this study, we confirmed the ability of the In2Care® Mosquito Traps to achieve all four of these criteria.

Gravid *Ae*. *aegypti* mosquitoes were attracted to the traps as observed by the number of positive traps and larvae collected from the traps. In the dry season, it took longer before most of the traps became positives for *Ae*. *aegypti* mosquito larvae, most likely because of an overall lower mosquito population abundance during this time of the year. The results also showed a relatively fast decline in water content in the dry season, whereby five of the traps (25%) would have been dry after six weeks indicating that during the dry season, the traps need to be revisited within 6 weeks to be refilled with water. If used in tropical areas, it could be recommended to refill the traps with water and insecticides every six weeks and every twelve weeks during the dry and rainy seasons, respectively. However, the efficacy of the adulticide (*Beauveria bassiana*) was not confirmed for the twelve-week period in this study. This should be confirmed before extending the recommendation for refill change every 12 weeks during rainy season.

It is known that PPF is a relatively stable compound and can usually persists for 2 months once added to water, depending on sunlight exposure and PPF dose [28]. In our study, the efficacy of PPF in the traps was still 100% after 12 weeks in field conditions. The presence of the lid on the traps is preventing exposure of PPF to sunlight, which is probably causing the prolonged efficacy. These results are similar to the field study of Su *et al*. [29] which showed a residual efficacy of the PPF in the traps after more than 29 weeks with a regular servicing every 3–4 weeks. Bukner *et al*. [30] compared the efficacy of the In2Care traps and an integrated vector management (source reduction, larviciding and adulticiding) in a six months large field trial in Florida. The traps were serviced every 4 months and the results showed a reduction of eggs, larvae and adults abundance in the area treated with the In2Care traps showing the potential of auto-dissemination in large areas. Furthermore, another explanation for the efficacy of the PPF on larval mortality and adult emergence is that juvenile hormone mimics such as PPF and *s*-methoprene, tend to be retained by plastic and render long residual activity [31]. Extended use under tropical conditions did increase the trap attractiveness to egg-laying *Aedes* mosquitoes and more than 75% of the traps were larvae-positive after 3 months and 100% of these larvae died due to PPF and/or spinosad. These findings indicate that the trap servicing time interval could be extended to at least 3 months in the rainy season regarding the PPF and spinosad residual efficacy.

In our study, the effect of *B*. *bassiana* on adult mortality was not studied. It is therefore not clear if the efficacy of the fungus was optimal in our test conditions. Some studies showed that the fungal infection can reduce mosquito survival in semi-field conditions by 59–95% in large cages [32] and is temperature dependant [33]. Buckner *et al*. [24] showed that under semi-field trial conditions the mosquitoes were able to pick up enough fungus to reduce their survivorship. However, their study also mentioned that during their experiment the temperature was low (22°C) which is far from the temperatures that can be recorded in Vientiane capital usually (>30°C in the dry season). Indeed, the optimal growth temperature for *B*. *bassiana* is isolate-dependent and can vary between 20°C and 30°C [33]. The efficacy of the fungus should be evaluated under the hot conditions in tropical areas to determine its residual efficacy.

The traps treated with both In2Mix® alone and In2Mix® with spinosad showed 100% of larval and pupal mortality in the two trials. This confirms that the mixture of the two active ingredients is considered to be a promising new control strategy for container-inhabiting *Aedes* [13, 28]. In Lao PDR, a previous study showed that Vientiane *Ae*. *aegypti* population was susceptible to PPF and spinosad [1]. Those insecticides were effective in a semi-field trial setting for more than 28 weeks in large water containers protected against sunlight [1]. When mixed together, the combination of spinosad and PPF also showed promising results in term of residual efficacy in large field trials in Martinique (French West Indies) because of their synergistic effect [13, 28]. The mixture also may have an advantage, regarding to the population perception of the treatment, due to the fast killing effect of spinosad on the field. Indeed, sometimes inhabitants are concerned with the presence of larvae in the traps and may not be aware of the efficacy of PPF. However, including spinosad might make the traps less cost-effective. Also, it would be interesting to study the attractiveness of the traps treated with spinosad compared to traps treated only with PPF. Indeed, the In2Care® mosquito Traps uses PPF, because larvae stay alive and attract more females to lay their eggs.

In2Care® Mosquito Traps were an effective tool for mosquito-driven larval control in water-filled containers in their nearby areas. During trap deployment, results from the buckets surveillance showed a 100% emergence inhibition. The quantities of PPF may be sufficient for effective larval control in small containers like the 5L bucket we used or the flower pots used in Buckner *et al*. [24], but it is unclear if PPF auto-dissemination can also be effective for larger larval habitats such as water storage containers used in Vientiane that can have volumes over than 50 liters. Therefore, future works are needed to determine if mosquitoes are able to pick up enough PPF from the stations to prevent the emergence of mosquitoes in environments with countless containers of all shapes and sizes. Indeed, there was only two water containers in the surrounding of the In2Care® Mosquito Traps and this is an important limitation of our study. The use of only one type of bucket and only two of them cannot demonstrates universal dissemination to surrounding habitats in real conditions. A recent large scale study in Amazonian Brazil [34] showed that mosquito PPF auto dissemination has potential to block arbovirus transmission city wide with significant reduction of *Aedes* juvenile catch and adult emergence, while *Aedes* juvenile showed high increased mortality (from 2%-7% to 80%-90%) as well as the number of females emerging per person decreased to 0.002–0.129 females per person-month.

The results of the BG-traps surveillance showed that the adult mosquito population abundance in the deployment area in the 2 trials were lower than before and after the years of implementation of In2Care® Mosquito Traps but it was not significant. This could be explained by the location of the BG traps that were placed at the periphery of the treated area (Fig 1) and the probable entry of adult mosquitoes from surroundings of the study area. In addition, the number of adults collected was very low throughout the years, which explains the low statistical power of the analysis. In combination with the In2Care® Traps, other methods such as source reduction or larval treatment could be used around the treatment area to act as a buffer for optimal protection.

## Conclusions

Since *Aedes spp*. mosquitoes may transmit viruses such as dengue, chikungunya and zika, the finding of our limited study that In2Care® Mosquito Traps can effectively lure *Ae*. *aegypti* females and kill their offspring in and around the traps indicate that this strategy should be tested in larger scale field trials to assess the efficacy in realistic conditions before being deployed by mosquito abatements and government public health campaigns. If efficient, in this conditions, this strategy could be used in selected risk areas in Lao PDR such as known dengue hotspots but also permanently in hospitals, schools, temples, hotels and markets.

The use of different actives in a mixture could preserve the utility of insecticides in public health programs regarding Insecticide Resistance Management, if this will still be cost effective.

## Supporting information

S1 Table Water levels and quality in the traps and their GPS coordinates. (XLSX)
